# Analytical Performances of Human Immunodeficiency Virus Type 1 RNA-Based Amplix® Real-Time PCR Platform for HIV-1 RNA Quantification

**DOI:** 10.1155/2016/7954810

**Published:** 2016-12-05

**Authors:** Christian Diamant Mossoro-Kpinde, Ralph-Sydney Mboumba Bouassa, Mohammad-Ali Jenabian, Serge Tonen Wolyec, Leman Robin, Mathieu Matta, Jean de Dieu Longo, Gérard Grésenguet, Laurent Andreoletti, Laurent Bélec

**Affiliations:** ^1^Laboratoire National de Biologie Clinique et de Santé Publique, Bangui, Central African Republic; ^2^Faculté des Sciences de la Santé, Université de Bangui, Bangui, Central African Republic; ^3^Laboratoire de Virologie, Hôpital Européen Georges Pompidou, Paris, France; ^4^Université Paris Descartes, Paris Sorbonne Cité, Paris, France; ^5^Département des Sciences Biologiques et Centre de Recherche BioMed, Université du Québec à Montréal (UQAM), Montreal, QC, Canada; ^6^Faculté de Médecine, Université de Bunia, Bunia, Democratic Republic of the Congo; ^7^Centre National de Référence des Maladies Sexuellement Transmissibles et de la Thérapie Antirétrovirale, Bangui, Central African Republic; ^8^Unité de Recherches et d'Intervention sur les Maladies Sexuellement Transmissibles et le SIDA, Département de Santé Publique, Faculté des Sciences de la Santé de Bangui, Bangui, Central African Republic; ^9^Laboratoire de Virologie Médicale et Moléculaire, EA-4684/SFR CAP-SANTE, Reims, France

## Abstract

*Objectives*. We evaluated the performances of Amplix real-time PCR platform developed by Biosynex (Strasbourg, France), combining automated station extraction (Amplix station 16 Dx) and real-time PCR (Amplix NG), for quantifying plasma HIV-1 RNA by lyophilized HIV-1 RNA-based Amplix reagents targeting* gag* and* LTR*, using samples from HIV-1-infected adults from Central African Republic.* Results*. Amplix real-time PCR assay showed low limit of detection (28 copies/mL), across wide dynamic range (1.4–10 log copies/mL), 100% sensitivity and 99% specificity, high reproducibility, and accuracy with mean bias < 5%. The assay showed excellent correlations and concordance of 95.3% with the reference HIV-1 RNA load assay (Roche), with mean absolute bias of +0.097 log copies/mL by Bland-Altman analysis. The assay was able to detect and quantify the most prevalent HIV-1 subtype strains and the majority of non-B subtypes, CRFs of HIV-1 group M, and HIV-1 groups N and O circulating in Central Africa. The Amplix assay showed 100% sensitivity and 99.6% specificity to diagnose virological failure in clinical samples from antiretroviral drug-experienced patients.* Conclusions*. The HIV-1 RNA-based Amplix real-time PCR platform constitutes sensitive and reliable system for clinical monitoring of HIV-1 RNA load in HIV-1-infected children and adults, particularly adapted to intermediate laboratory facilities in sub-Saharan Africa.

## 1. Introduction

The increased availability of antiretroviral treatment (ART) for HIV infection has resulted in major reductions in morbidity and mortality in high HIV burden with poor resources settings. Through significant global scale-up, access to ART is increasing with more than 15 million people in low-income settings by the end of 2015 which represent around 65% of the global target [1].

In 2013, the World Health Organization (WHO) published first consolidated guidelines on the use of ART for HIV infection across all age groups and populations. The 2013-revised WHO guidelines for scaling up ART in adults and children living in resource-limited settings emphasized the need of laboratory monitoring, first based on immunological assessment of CD4 T-cell count, mainly to start ART and monitor patients on ART, and secondly based on the plasma HIV-1 RNA load in order to monitor treatment efficacy and early therapeutic failure as well as to monitor therapeutic switches [2–5]. More recently, the ambitious UNAIDS Fast-Track targets for 2020, including achievement of major reductions in HIV-related mortality and new HIV infections and the 90–90–90 targets, pushed countries to further accelerate their actions against HIV epidemics in upcoming years. A comprehensive revision of the consolidated WHO guidelines on the use of ART has been undertaken in 2015 based on new scientific evidence and lessons from antiretroviral programs implementation [1]. Basically, the key recommendation made in 2015 is the fact that ART should be initiated in everyone living with HIV at any CD4 T-cell count as this may result in a better clinical outcome [1].

In 2016, the HIV-1 RNA load constitutes the principal biological marker to monitor ART efficacy and early therapeutic failure [1]. Sensitive and accurate measurements of HIV-1 viral load are critical for clinical management and monitoring of ART. Several nucleic acid-based technologies have been already developed for quantification of HIV viral RNA [6]. The complicating factors that characterize HIV RNA viral load assays and platforms, which lead to the choice of platforms for a given setting, include HIV diversity including HIV-1 subtypes and circulating recombinant forms and certain practical challenges, such as laboratory infrastructures and samples transport. However, the great majority of testing options available today for HIV RNA load remains sophisticated laboratory-based platforms with nucleic acid-based assays performed on complex instrumentation requiring dedicated laboratory space and trained laboratory technicians [6–8]. Despite the clinical consensus on the importance of viral load testing particularly for detection of treatment failure, routine measurement of plasma HIV-1 RNA load remains difficultly affordable and unsustainable in a great majority of resource-limited settings, notably in South Africa [7].

Recently, intermediate high-throughput laboratory platforms for HIV-1 RNA load have emerged [6]. Intermediate viral load platforms are expected to overcome some of the technological limitations to improve viral load assessment as well as reduction of costs and may be particularly adapted for categories III (i.e., intermediate) and IV (i.e., reference) laboratories, according to the 2008 Maputo classification of laboratory facilities [9]. Recently, Biosynex (Strasbourg, France) has developed a new real-time RT-PCR-based platform for HIV-1 RNA quantification, combining a fully automated station for nucleic acids extraction (Amplix station 16 Dx, Biosynex) and real-time PCR amplification (Amplix NG, Biosynex) and using lyophilized HIV-1 RNA-based Amplix real-time PCR kit (Biosynex) targeting* gag* and* LTR* genes.

In the present report, we evaluated the HIV-1 RNA-based Amplix real-time PCR platform according to the Clinical and Laboratory Standards Institute (CLSI) guidelines for clinical use [10, 11] and guidelines recommended by the pSMILE project (Patient Safety Monitoring in International Laboratories) (http://www.psmile.org/), in Bangui, the capital of the Central African Republic, a country of broad genetic diversity and high prevalence of non-B subtypes of HIV-1 group M.

## 2. Material and Methods

### 2.1. Standards

Two standard WHO panels of HIV-1 were used to study the analytical performances of the assay. The 2010-established 3rd WHO HIV-1 international standard (National Institute for Biological Standards and Control, Potters Bar, Hertfordshire, UK; NIBSC code: 10/152) is based on HIV-1 subtype B field isolate, isolated postmortem from a patient who died from AIDS-defining illness, propagated on PBMCs allowing low passage stock stored down. This 3rd WHO HIV-1 standard has been assigned a value of 185,000 international units (IU)/mL [i.e., 111,000 copies/mL, 5.04 log copies/mL, when using the conversion factor 0.58 copies/IU [12]]. The 2000-established 2nd WHO international reference panel preparation of various HIV-1 subtypes (National Institute for Biological Standards and Control; NIBSC code: 12/224) consists in a well-characterized reference panel of 10 different HIV-1 groups, including group M subtypes A, B, C, D, AE, F, G, and AA-GH, group N, and group O. The AcroMetrix® HIV-1 panel (applied Biosystems, AcroMetrix Corporation, Benicia, CA, USA) consists of HIV-1 standards at concentrations of 10,000,000, 1,000,000, 100,000, 10,000, and 1,000 IU/mL calibrated against the WHO international HIV-1 RNA standard.

### 2.2. Clinical Specimens

The so-called* Centre National de Référence des Maladies Sexuellement Transmissibles et de la Thérapie Antirétrovirale* constitutes an open health care center for ambulatory adults infected by HIV in Bangui, the capital city of the Central African Republic. The active file comprised in 2014 around 2,500 patients, including 850 treated by ART according to the WHO 2013-revised recommendations for resource-poor settings [13]. All HIV-1-infected patients were prospectively included on the basis of the following criteria: age more than 18 years, signed informed consent, and ART for more than 6 months. HIV-negative patients consulting for sexually transmitted infection were also recruited as negative control. For all patients, blood samples were collected by venipuncture using tripotassium EDTA- (K_3_-EDTA-) tubes and cells separation was processed to obtain plasma within 4 hours after collection. Surplus plasma aliquots were stored frozen (−80°C) until testing. One plasma aliquot was further transported on dry ice to the virology laboratory of the Hôpital Européen Georges Pompidou (HEGP) for possible genotypic resistance test.

### 2.3. Ethical Statement

The study has been formally approved by the Scientific Committee of the Faculté des Sciences de la Santé (FACSS) of Bangui (so-called Comité Scientifique de Validation des Protocoles et Résultats de Recherche/CSVPR) (agreement UB/FACSS/CSVPR), which constitutes the National Ethics Committee.

### 2.4. HIV-1 RNA Quantification Assays

#### 2.4.1. Amplix Extraction, Real-Time PCR Amplification, and HIV-1 RNA Quantitative Kit

**Figure 1:**
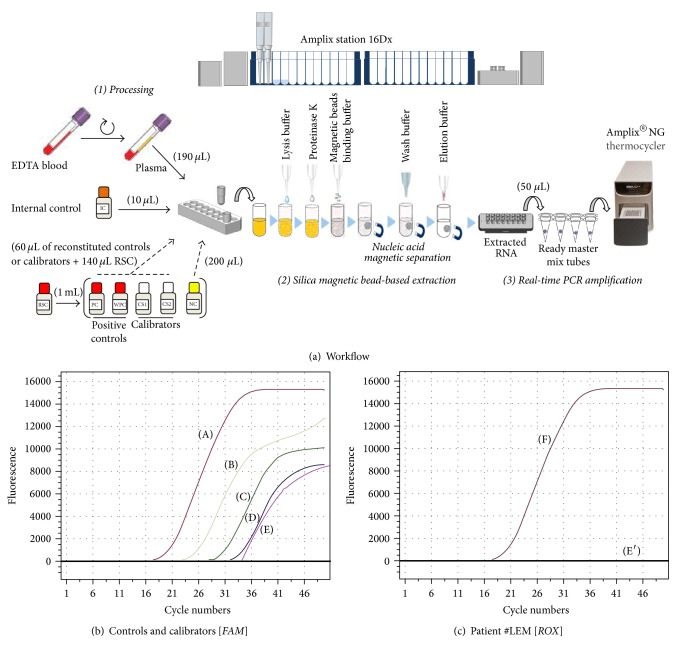
(a) Workflow for HIV-1 RNA load measurement using the Amplix platform combining automated station for nucleic acids extraction (Amplix station 16 Dx, Biosynex) and real-time PCR amplification (Amplix NG, Biosynex) and lyophilized HIV-1 RNA-based Amplix real-time PCR kit (Biosynex) targeting* gag* and* LTR* genes. (1) Samples preparation and preextraction processing, 190 *µ*L of plasma, positive control (PC), weak positive control (WPC), negative control (NC), and calibration samples (CS) 1 and CS2, in which 10 *µ*L of internal control (IC) is added, are subjected to DNA/RNA extraction, using the Amplix viral extraction kit (Biosynex). (2) Automated silica magnetic bead-based extraction (92 min). (3) Real-time PCR amplification (2 h 30) of extracted nucleic acids in 50 *µ*L of elution buffer is deposited in ready master mix tubes containing all reagents for reverse transcription and PCR. RSC: recovery solution for control samples. (b) Typical amplification curves of controls and calibrators given by the Amplix DTmaster software (Biosynex) showing the duplex detection of fluorescence labelled hydrolysis probes (5′-fluorescein carboxylic acid [FAM] and 3′-black hole quencher-1 [BHQ1]) of PC (A), CS1 (B), CS2 (C), WPC (D), and NC (E), giving Ct values of 16.0, 21.3, 26.6, 30.4, and 33.0, respectively. (c) Amplification curves given by the Amplix DTmaster software (Biosynex) showing the duplex detection of fluorescence labelled hydrolysis probes (5′-carboxy-rhodamine-X [ROX] and 3′-black hole quencher-2 [BHQ2]) of the HIV-1-infected Patient #LEM (F) showing Ct at 36.7 and plasma HIV-1 RNA load of 7,700 copies/mL and of the NC (E′).

Plasma HIV-1 RNA load was carried out using the Amplix platform developed by Biosynex (Strasbourg, France), which integrates a fully automated station for nucleic acids extraction (RNA and/or DNA) and real-time PCR amplification station (Figure 1(a)), using lyophilized Amplix HIV-1 RNA quantitative reagents (Biosynex).

The Amplix station 16 Dx is an automated nucleic acid extraction and purification instrument based on RNA/DNA attachment to magnetic silica particles, with further elution using chaotropic agent. The Amplix station 16 Dx is able to handle from 1 up to 16 clinical samples at the same time, while also offering a refrigerated platform at +4°C for preservation of extracted nucleic acids after each run and diagnostic kit integrity and providing an automatic pipetting function for true hands-off operation. To prevent any contamination, the Amplix station 16 Dx combines ultraviolet light, a contamination shield to prevent liquid dripping, single-sample loading tubes to prevent cross-contamination, and a tip barrier to prevent convection-induced aerosol formation. The Amplix viral extraction kit (Biosynex) for DNA/RNA extraction integrates prefilled buffer cartridge system containing all buffers, consumables, and enzymes necessary for successful reaction in 90 minutes per run. Extracted nucleic acids were finally eluted in 50 *μ*L of elution buffer.

The Amplix HIV-1 quantitative real-time PCR kit (Biosynex) targeting* gag* and* LTR* genes was used for detecting and quantifying HIV-1 RNA, according to the manufacturer's instructions. During the preextraction processing, 10 *μ*L of internal control (IC) is added to each plasma sample (190 *μ*L), positive control (PC), weak positive control (WPC), negative control (NC), and, at first run of the kit, calibration samples (CS) 1 and CS2. The IC consists of mRNA of the human gene for GAPDH (glyceraldehyde-3-phosphate dehydrogenase). The IC provides control of the whole amplification process, including sample RNA degradation (i.e., sample quality), efficiency of RNA extraction, and efficiency of reverse-transcription step and subsequent PCR amplification (i.e., PCR inhibition checking). For HIV-1 RNA quantification, a premix mixture was constituted by combining 50 *μ*L of eluate and lyophilized PCR reagents. After sealing, the dedicated PCR tubes were mixed thoroughly by using minispin centrifuge to dissolve the premix pellet with the nucleic acid extracts and then subjected to amplification using real-time thermocycler Amplix NG (Biosynex). The Amplix NG uses thermal blocks in 48-well format adapted to microplates, tubes, and strips, light emitting diodes (LED) as a source of light (life-time of about 100,000 hours), and 4 channels of fluorescence detection for detection of HEX, FAM, CY5, and ROX fluorescence dyes. The Amplix DTmaster software (Biosynex) controls the Amplix NG thermocycler and is used to depict and interpret final results of real-time PCR. The software may be used easily with intuitive control, allowing significant reduction of operator's time expenditure on creating protocols and minimizing risk of errors. The assay used dUTP instead of dTTP in the oligodeoxyribonucleotide primers, avoiding carry-over contamination of PCR products by uracil DNA glycosylase (UDG) decontamination, followed by thermal inactivation of UDG. To obtain precise measurement of viral HIV-1 RNA concentration in analyzed samples, calibration is needed at first run using CS1, CS2, and PC whose HIV-1 RNA concentrations are given in the passport of the kit, and the resulting values are registered for further runs with the same kit. The thermocycler has protection from interruptions of network power supply (with automatic resuming of amplification program following the restoration of network power supply) and from computer failures (with automatic resuming of device operation in case of computer problems with automatic reading of results of optical measurements from the device to computer following their elimination). Finally, for HIV-1 RNA quantification, the reaction mixture was subjected to UDG decontamination for 2 min at 37°C, reverse transcription during 5 min, and then to 20 sec of initial denaturation at 95°C and 50 PCR cycles, each of 10 sec at 94°C and 20 sec at 60°C, followed by a final cooling step of 10 min at 10°C.

The Ct (cycle threshold) value for which PCR was defined as positive was less than 45. The IC detection used 2 channels capturing the fluorescence issued from hydrolysis probes (5′-fluorescein carboxylic acid [FAM] and 3′-black hole quencher-1 [BHQ1]). The HIV cDNA detection (plasma samples, PC, WPC, CS1, and CS2) used 2 channels capturing the fluorescence issued from hydrolysis of two other probes (5′-carboxy-rhodamine-X [ROX] and 3′-black hole quencher-1 [BHQ2]). The NC should show only FAM fluorescent signal increase and no significant ROX fluorescent increase (Ct value for NC along ROX channel less than 40 indicating contamination) (Figures 1(b) and 1(c)). For a given plasma sample, the Amplix DTmaster software (Biosynex) detects the amplification signal increase for IC cDNA (FAM channel), which is valid if Ct of IC for this sample is equal to or less than 40, and the fluorescence signal increase for HIV cDNA (ROX channel). The HIV-1 RNA load for a given sample k calculated by Amplix DTmaster software is function of a B coefficient determined during initial calibration, the HIV-1 RNA load of the PC (specified in the passport of the kit), Ct of PC and Ct and sample k on ROX channel, and Ct of IC of PC and Ct of IC of sample k on FAM channel.

*Nota Bene*. HIV-1 RNA-based Amplix real-time PCR assay is stored at +2–8°С for 12 months after production. However, transportation at +25°С for 10 days is allowed. The reagents should not be frozen. The ready master mix tubes for reverse transcription and PCR [96 test tubes (12 strips × 8 tubes) per kit] are stored freeze-dried and are ready to use after adding the 50 *µ*L of extract eluate. Control samples (PC and WPC) and CS are stored freeze-dried and are reconstituted by adding 1 mL of recovery solution for control samples (RSC) into each well which should be stored at +2–8°С and used within 1 month after preparation. A total of 60 *µ*L of reconstituted PC, WPC, and CS are mixed with 140 *µ*L of RSC before extraction. The specific HIV-1 RNA concentrations of PC and WPC are indicated on a dedicated passport within the kit. The IC consisting of GAPDH (glyceraldehyde-3-phosphate dehydrogenase) mRNA in buffer is ready to use and stored at +2–8°С. Negative controls consisted of RNA extracted from plasma of healthy subject.

#### 2.4.2. COBAS AmpliPrep/COBAS TaqMan HIV-1 Roche Assay

The COBAS AmpliPrep/COBAS (CAP/CTM®) TaqMan HIV-1 test v2.0 (Roche Molecular Diagnostics, Basel, Switzerland) is a quantitative RT-PCR assay amplifying a conserved region in* gag* and* LTR* regions of HIV-1 using a TaqMan probe. The COBAS TaqMan uses an internal control simultaneously extracted and amplified with each sample. The assay detects HIV-1 groups M and O and several CRFs [14]. The viral load determination was performed using the COBAS AmpliPrep nucleic acid extractor from 1 mL of plasma; the amplification and real-time detection were carried out using COBAS TaqMan 48 thermocycler. The assay dynamic range is 1.6 to 7.0 log copies/mL of plasma samples. The CAP/CTM HIV-1 test v2.0 was chosen as reference assay for HIV-1 RNA load quantification in present study.

#### 2.4.3. HIV-1 Genotyping

A genotypic resistance test was carried out for a subgroup of HIV-treated patients whose HIV-1 RNA load was above 1,000 copies/mL. Genotypic analyses of reverse transcriptase and protease HIV-1 genes were performed at the virology laboratory of HEGP, Paris, France, with the commercial assay ViroSeq® HIV-1 genotyping system v2.0 (Celera Diagnostics, CA, USA) used on plasma samples. The virology laboratory of HEGP is accredited by the Comité Français d'Accréditation (COFRAC) regarding the exigence of the ISO 15189 norm for the biological markers HIV RNA load and antiretroviral drug resistance genotype. The resulting* pol* sequences were aligned using ViroSeq HIV-1 genotyping system software v2.6 (Celera Diagnostics). HIV-1 subtype was assessed using the resulting pol sequences with the genotyping software (National Center for Biotechnology Information; https:// www.ncbi.nlm.nih.gov/projects/genotyping/formpage.cgi), as described previously [15].

### 2.5. Analytical Performances of HIV-1 RNA-Based Amplix Real-Time PCR

#### 2.5.1. Lower Limit of Detection and Linear Range

To determine the lower limit of detection, HIV-1 standards were prepared at 100, 75, 50, 40, 30, 20, and 10 HIV-1 RNA copies/mL by serial dilution in HIV-negative plasma of the 3rd WHO HIV-1 subtype B international standard (National Institute for Biological Standards and Control), to be further tested 37 to 65 times by HIV-1 RNA-based Amplix real-time PCR assay. The assay linear range of the assay was determined using serial dilutions in HIV-1­negative human EDTA plasma from a highly concentrated cell culture stock of HIV-1 group M subtype B measured by CAP/CTM HIV-1 test v2.0 (Roche Molecular Systems) at 10,001,789 copies/mL prepared in HIV-seronegative EDTA plasma.

#### 2.5.2. Sensitivity and Specificity

The sensitivity and specificity of the HIV-1 RNA-based Amplix real-time PCR assay were assessed by testing clinical specimens from 215 HIV-1-infected patients with detectable HIV-1 RNA load by the CAP/CTM HIV-1 test v2.0 and 103 clinical specimens from HIV-1-seronegative individuals with undetectable HIV-1 RNA load by the CAP/CTM HIV-1 test v2.0.

#### 2.5.3. Repeatability and Reproducibility

In addition to the use of the 3rd WHO HIV-1 subtype B international standard (National Institute for Biological Standards and Control), two pools of 30 plasmas were constituted: the low-positive pool (LPP) with a resulting HIV-1 RNA load at 1,513 copies/mL (3.17 log/mL) and the high-positive pool (HPP) with a resulting HIV-1 RNA load at 2,150,033 copies/mL (6.33 log/mL). To assess repeatability, the 3rd WHO standard and the LPP and HPP were tested 3 and 30 times, respectively, at the same day, by the same technician using the same batch of Amplix real-time PCR reagents. To assess interassay reproducibility, the 3rd WHO standard and the LPP and HPP were tested 3 and 30 times, respectively, in corresponding runs on different days, during a 3-week period, by different technicians using different batches of Amplix reagents. The resulting coefficients of variation (CV) % = (standard deviation/mean) × 100 were calculated.

#### 2.5.4. Estimation of the Accuracy

The assay accuracy was evaluated by preparing standards at nominal concentrations of 20, 100, 1,000 (threshold of viral failure according to 2013-revised WHO criteria), 5,000, and 50,000 HIV-1 RNA copies/mL from the AcroMetrix HIV-1 panel (applied Biosystems) and quantifying HIV-1 RNA levels in each sample five times the same day by the HIV-1 RNA-based Amplix real-time PCR assay. Each sample was carried through the entire Amplix platform procedure, including specimen preparation, amplification, and detection operated by multiple users. Therefore, the estimation of the accuracy represents all aspects of the test procedure. The resulting bias (%) was calculated for each nominal standard.

### 2.6. Influence of the HIV-1 Genotypes

HIV-1 genetic diversity could affect the performance of viral load assays [16–18], especially in sub-Saharan Africa, where non-B subtypes HIV-1 largely predominate [19]. The influence of HIV-1 genotypes was assessed (i) by the HIV-1 group and subtype limit of detection of various genotypes of HIV-1 contained in the 2nd WHO international reference panel and (ii) by the quantification of HIV-1 RNA in clinical samples containing broad diversity of HIV-1 group M subtypes with reference to the quantification by the CAP/CTM HIV-1 test v2.0.

### 2.7. Evaluation of Contamination between Samples

To assess the intersamples contamination, the HPP was tested consecutively 3 times (H1, H2, and H3; mean, mH), followed by the testing of LPP consecutively 3 times (L1, L2, and L3), (B1, B2, and B3), and the sequences (H1, H2, H3, L1, L2, and L3) were repeated 5 times, and the resulting means of L1 (mL1) and of L3 (mL3) were calculated. The intersamples contamination C was calculated according to the following formula: C (%) = (mL1 – mL3)/(mH − mL3) × 100.

### 2.8. Statistical Analyses

All viral load results were reported in log copies/mL. The conversion factor applied was 0.58 copies per IU, as previously proposed [12, 20, 21]. Descriptive statistics are shown as the mean ± standard deviation (SD). Summary statistics are presented as estimates (95% confidence intervals [CI]).

The raw data were entered into an Excel spreadsheet. The Method Validator software, version 1.1.9.0. (Philippe Marquis, France) and the informatic facilities developed by BiostaTGV (*Institut Pierre Louis d'Epidémiologie et de Santé Publique UMR S 1136, INSERM and University Pierre et Marie Curie*, Paris, France; available at: http://marne.u707.jussieu.fr/biostatgv/) were used for statistical analyses.

The lower limit of detection was determined by PROBIT regression analysis (SPSS version 18, Chicago, IL).

Correlations between the viral load values obtained by the reference CAP/CTM HIV-1 test v2.0 and the HIV-1 RNA-based Amplix real-time PCR assay were established by unweighted linear regression and by the Passing-Bablok nonparametric regression which is fairly little sensitive to outliers [22]. The level of significance for Passing-Bablok correlations was set at *P* < 0.05.

The agreement between the reference CAP/CTM HIV-1 test v2.0 and the HIV-1 RNA-based Amplix real-time PCR assay was depicted by difference plots as proposed by Bland and Altman [23, 24]. The Bland-Altman analysis examines, in a discriminative fashion, whether the methods agree sufficiently well to be used interchangeably. The average of values obtained by the 2 methods is displayed on the *x*-axis and plotted against the difference between the 2 methods shown on the *y*-axis. The average difference between the 2 methods, referred to as bias, was calculated, respectively, as the mean *μ* and the SD of the difference between both methods, and marked on the graph by a horizontal line, and the limits of agreement with 95% CI were also calculated as *μ*  ±  1.96 SD and depicted.

Significant difference between two paired viral load results was taken above 0.5 log.

The concordance between HIV-1 RNA-based Amplix real-time PCR assay and reference CAP/CTM TaqMan HIV-1 test v2.0 was estimated by Cohen's *κ* coefficient corresponding to the number of viral loads below or above the threshold of positivity by the reference assay correctly detected by the HIV-1 RNA-based Amplix real-time PCR, divided by the total number of viral loads correctly detected plus the false results, multiplied by 100 [25]; the accuracy of HIV-1 RNA-based Amplix real-time PCR assay to discriminate HIV-1 RNA positive and negative samples was estimated by Youden's *J*  index (*J* = sensitivity + specificity − 1).

For clinical significance of using the HIV-1 RNA-based Amplix real-time PCR assay on virological failure diagnosis, the efficiency of the Amplix assay to accurately discriminate between HIV-infected patients in virological failure according to the WHO threshold (i.e., >1,000 copies/mL) was estimated by Cohen's *κ* coefficient corresponding to the number of viral loads below or above the threshold of virological failure correctly detected by the HIV-1 RNA-based Amplix real-time PCR, divided by the total number of viral loads correctly detected plus the false results, multiplied by 100; the accuracy of HIV-1 RNA-based Amplix real-time PCR assay to diagnose virological failure was estimated by Youden's *J* index.

The confidence interval for all variables was calculated at 95% (95% CI) using a normal distribution.

## 3. Results

### 3.1. Analytical Performances of HIV-1 RNA-Based Amplix Real-Time PCR

#### 3.1.1. Lower Limit of Detection and Linear Range

**Figure 2:**
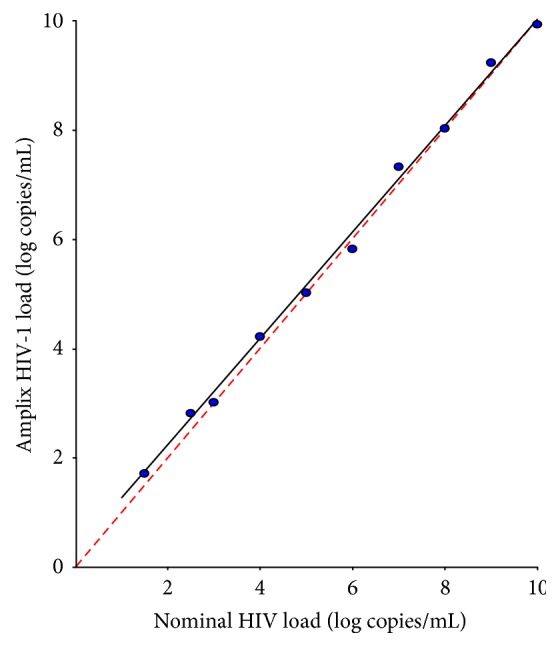
Linear range of the HIV-1 RNA-based Amplix real-time PCR assay. Viral HIV-1 RNA levels were measured in HIV-seronegative plasma samples that were spiked with HIV-1 group M subtype B viral stock (at 10,001,789 copies/mL) at final concentrations of 25, 50, 100, 500, 5000, 100,000, 500,000, 2,000,000, 5,000,000, and 10,000,000 copies/mL. Slope and Y-intercept values for the resultant trend line were 0.980 (95% CI, [0.934–1.026]) and 0.253, respectively, as determined by unweighted linear regression (*r* ^2^ = 0.981). Each sample was assayed in duplicate. The diagonal dotted line represents the ideal line (no bias). The full line represents the regression line of the distribution (*n* = 10).

The manufacturer claims that the lower limit of detection of the HIV-1 RNA-based Amplix real-time PCR assay is 20 copies/mL. The sensitivity of detection was 100% for RNA concentrations ≥ 50 copies/mL (Table 1). Based on PROBIT regression analysis, the mean lower limit of detection for the assay was 28.1 HIV-1 RNA copies/mL (95% CI, 20.1–82.8 copies/mL). A linear response was consistently observed across the entire range of the HIV-1 group M subtype B viral stock dilutions tested (nominal 25 ≥ 10,000,000 copies/mL) (Figure 2).

#### 3.1.2. Sensitivity and Specificity

Of the 215 HIV-seropositive samples, they all tested positive for HIV-1 RNA by the HIV-1 RNA-based Amplix real-time PCR assay (sensitivity, 100.0%; 95% CI [99.7%–100%]). Of the 103 HIV-seronegative specimens, they all, but one weakly positive for HIV-1 RNA at 21 copies/mL, tested negative for HIV-1 RNA by the HIV-1 RNA-based Amplix real-time PCR assay (specificity, 99.0%; 95% CI [97.1%–100%]). Cohen's *κ* coefficient between both techniques was 0.99, demonstrating excellent concordance. Youden's *J* index of HIV-1 RNA-based Amplix real-time PCR assay was 0.99, demonstrating high efficiency to discriminate HIV-1 RNA positive and negative samples.

#### 3.1.3. Repeatability and Reproducibility

As shown in Table 2, the intra-assay coefficients of variation (repeatability) and interassay coefficients of variation (reproducibility) ranged from 4.6% to 7.7% and from 6.0% to 9.1%, respectively.

#### 3.1.4. Estimation of the Accuracy

As shown in Table 3, the calculated bias ranged from 2.6% to 4.6%, with a mean bias of 3.3%.

#### 3.1.5. Correlation and Agreement with Reference Method

**Figure 3:**
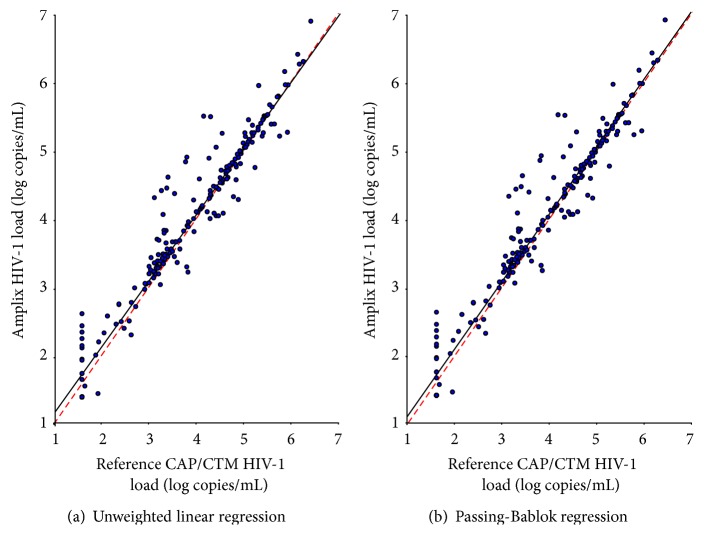
Unweighted linear regression (a) and Passing-Bablok regression (b) tests between plasma HIV-1 RNA viral load measurements in 215 HIV-1-infected adults, expressed in log copies/mL obtained in parallel by the HIV-1 RNA-based Amplix real-time PCR assay (Biosynex) and the reference COBAS® AmpliPrep/COBAS TaqMan (CAP/CTM) HIV-1 test v2.0 (Roche Molecular Diagnostics). By unweighted linear regression, slope and Y-intercept values for the resultant trend line were 0.968 (95% CI, [0.933–1.002]) and 0.219 (95% CI, [0.080–0.357]), respectively. By Passing-Bablok regression, slope and *Y*-intercept values for the resultant trend line were 0.987 (95% CI, [0.968–1.009]) and 0.102 (95% CI, [0.009–0.186]), respectively. The diagonal dotted lines represent the ideal lines (no bias). The full lines represent the regression lines of the distribution.

**Figure 4:**
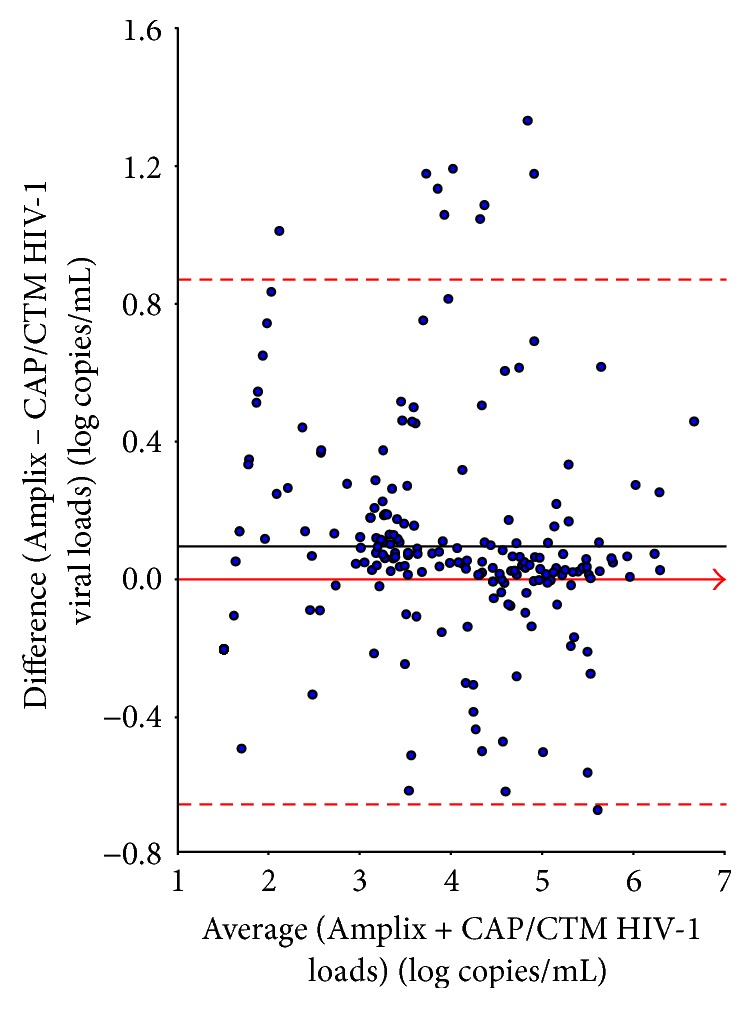
Bland-Altman agreement on the relative differences between the plasma HIV-1 RNA viral load measurements in 215 HIV-1-infected adults obtained in parallel by the HIV-1 RNA-based Amplix real-time PCR assay (Biosynex) and the reference COBAS AmpliPrep/COBAS TaqMan (CAP/CTM) HIV-1 test v2.0 (Roche Molecular Diagnostics). The full line represents the mean relative difference, and the horizontal dotted lines represent the superior and inferior limits of agreement [i.e., the mean differences ± 1.96 SD (SD = 0.377), representing the 95% confidence limits of the agreement]. There were 10 (4.6%) discrepant samples outside the 95% CI level of agreement between the results by HIV-1 RNA-based Amplix real-time PCR assay (Biosynex) and the reference COBAS AmpliPrep/COBAS TaqMan (CAP/CTM) HIV-1 test v2.0. The arrow corresponds to the *x* abscise axis.

A total of 215 plasma samples from HIV-infected patients were analyzed in parallel. HIV-1 RNA-based Amplix real-time PCR assay detected HIV-1 RNA in 198 (92.1%) samples and the reference CAP/CTM TaqMan HIV-1 test v2.0 in 187 (86.9%) samples (NS). The accuracy of HIV-1 RNA load measurements by HIV-1 RNA-based Amplix real-time PCR assay was carried out against the results obtained by the CAP/CTM TaqMan HIV-1 test v2.0 chosen as reference assay for plasma HIV-1 RNA load. The unweighted linear regression and Passing-Bablok regression of the results obtained by the HIV-1 RNA-based Amplix real-time PCR assay with reference to the CAP/CTM TaqMan HIV-1 test v2.0 are depicted in Figure 3.  Figure 4 depicts the Bland-Altman agreement analyses between the HIV-1 RNA load results obtained by HIV-1 RNA-based Amplix real-time PCR assay and CAP/CTM TaqMan HIV-1 test v2.0, in the 215 study HIV-seropositive plasma samples from antiretroviral drug-experienced patients. Mean ± SD of HIV-1 RNA load expressed in log copies/mL was 3.77 ± 1.28 (range, 1.60–6.42) by CAP/CTM TaqMan HIV-1 test v2.0 and 3.88 ± 1.30 (range, 1.39–6.88) by HIV-1 RNA-based Amplix real-time PCR assay (*P* > 0.5). The nonparametric Passing-Bablok regression analysis on all 215 available HIV-1 RNA load results revealed a high correlation between plasma HIV-1 RNA load obtained by HIV-1 RNA-based Amplix real-time PCR assay and CAP/CTM TaqMan HIV-1 test v2.0 with a slope of 0.98 and an intercept of +1.02. The relation between HIV-1 RNA-based Amplix real-time PCR assay and CAP/CTM TaqMan HIV-1 test v2.0 did not differ from linearity (*P* > 0.4). The mean absolute bias measured by Bland-Altman analysis between HIV-1 RNA load obtained by HIV-1 RNA-based Amplix real-time PCR assay and CAP/CTM TaqMan HIV-1 test v2.0 over the entire range of plasma HIV-1 RNA load results was +0.097 log copies/mL (95% CI: +0.052–0.142) with limits of agreement from −0.657 to +0.851 log copies/mL. Thus, the HIV-1 RNA-based Amplix real-time PCR assay was quantified significantly higher than CAP/CTM TaqMan HIV-1 test v2.0 (*P* = 0.001). Overall, ≥95% (205/215) of the paired HIV-1 RNA load results fell within the 95% CI levels of agreement when compared with the PCR assays by Bland-Altman analysis. Of these 10 discrepant samples, 9 showed difference of more than +1 log copies/mL (2 CRF06-cpx, 2 CRF01_AE, 3 CRF01_AG, and 2 CRF15_01B) and 1 of more than −1 log copies/mL (1 subtype G).

### 3.2. Influence of the HIV-1 Genotypes

#### 3.2.1. Limits of Detection of Various Genotypes of HIV-1

One dilution series (15 to 30 copies/mL) of the different HIV-1 genotypes contained in the 2nd WHO international reference panel were evaluated on two days with one lot of the Amplix real-time PCR assay in a total of 10 replicates per concentration level for each genotype of HIV-1. The results of the PROBIT analyses at 95% rate demonstrate that the Amplix real-time PCR assay showed a sensitivity of ≤50 copies/mL across all HIV-1 genotypes tested ranging from 19 to 29 copies/mL of HIV-1 group M, 27 copies/mL of HIV-1 group N, and 24 copies/mL of HIV-1 group O, as shown in Table 4.

#### 3.2.2. Quantification of HIV-1 RNA in Clinical Samples Containing Broad Diversity of HIV-1 Group M Subtypes

**Figure 5:**
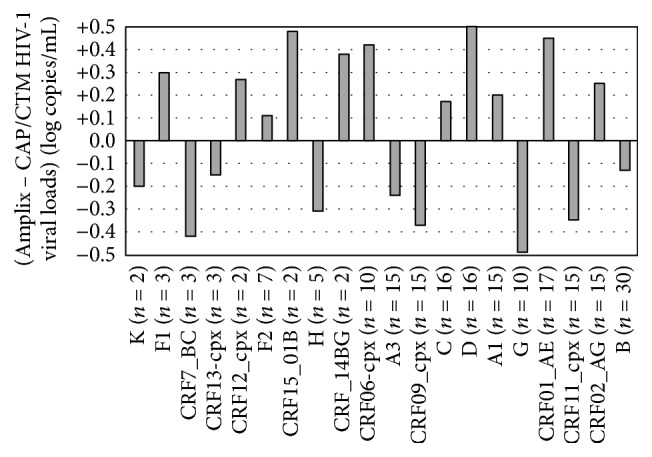
Mean differences (log copies/mL) of HIV-1 RNA loads measured in parallel by HIV-1 RNA-based Amplix real-time PCR assay (Biosynex) and by the reference COBAS AmpliPrep/COBAS TaqMan HIV-1 test v2.0 (Roche Molecular Diagnostics) in clinical plasma samples from 168 patients receiving antiretroviral regimen and in virological failure, representing a broad series of HIV-1 group M B subtype and non-B subtypes. Significant differences between two paired HIV-1 RNA load results were taken above 0.5 log.

A total of 168 plasma samples from patients receiving antiretroviral regimen having virological failure (i.e., viral load > 1,000 copies/mL) were selected for drug resistance mutations genotyping and HIV-1 subtype assessment from the resulting protease or reverse transcriptase* pol* gene sequences. HIV-1 from the series of plasma samples showed broad series of HIV-1 group M subtypes [including the following subtypes: B, CRF02_AG, CRF11_cpx, CRF01_AE, G, A1, D, C, CRF09_cpx, A3, CRF06-cpx, CRF_14BG, H, CRF15_01B, F2, CRF12_cpx, CRF13-cpx, CRF7_BC, F1, and K]. Plasma HIV-1 RNA loads were then measured in parallel by HIV-1 RNA-based Amplix real-time PCR assay and by the reference CAP/CTM HIV-1 test v2.0, and the mean differences of viral loads measured by both assays were determined for each HIV-1 subtype.  Figure 5 shows that the mean differences of HIV-1 RNA load by the Amplix real-time PCR assay and the reference Roche assay were always below 0.5 log for all HIV-1 subtypes analyzed, demonstrating similar capacity of both assays of detecting HIV-1 B and non-B subtypes from clinical samples.

### 3.3. Assessment of Lack of Contamination between Samples

The intersamples contamination C was 0% (not shown).

### 3.4. Capability of Detecting Virological Failure in Clinical Samples

Of the 215 HIV-seropositive plasma samples from antiretroviral drug-experienced patients, 168 and 170 showed HIV-1 RNA load above 1,000 copies/mL, the WHO threshold of virological failure (WHO, 2013), by CAP/CTM HIV-1 test v2.0, and HIV-1 RNA-based Amplix real-time PCR assay, respectively (Table 5). Thus, the sensitivity of HIV-1 RNA-based Amplix real-time PCR assay to diagnose virological failure was 100% (95% CI, [99.7%–100%]) and the specificity 99.6% (95% CI [89.9%–100%]). Cohen's *κ* coefficient between both techniques was 0.97, demonstrating their excellent concordance to diagnose virological failure. Youden's *J* index of HIV-1 RNA-based Amplix real-time PCR assay was 0.96, demonstrating high efficiency to discriminate samples with viral load < 1,000 copies/mL and those in virological failure.

## 4. Discussion

Plasma HIV-1 RNA load is a routine investigation for monitoring of HIV-1-infected individuals. We herein evaluated the performances of plasma HIV-1 RNA measurement by the new HIV-1 RNA-based Amplix real-time PCR platform developed by Biosynex. The analytical performance characteristics of the Amplix HIV-1 quantitative real-time PCR kit were quite similar to those usually obtained with commercial HIV RNA assays. Thus, the detection cutoff of 28 copies/mL was close to cutoffs generally reached with other commercial assays. The linearity range from 25 to 10 million copies/mL allows only one test in case of high viral load. The assay was highly sensitive and specific to detect HIV-1 RNA, showing excellent concordance with reference assay. This assay was highly reproducible with good intra- and interrun below 15%, as recommended for nucleic acid-based technologies [6]. The Amplix assay was highly accurate with a mean bias below 5%. Furthermore, excellent correlations were obtained between Amplix real-time PCR test results and those obtained by the reference CAP/CTM TaqMan HIV-1 test v2.0 (Roche Molecular Diagnostics). The Amplix assay was highly sensitive, specific, and accurate to diagnose virological failure (i.e., viral load > 1,000 copies/mL) in clinical samples from antiretroviral drug-experienced patients, demonstrating excellent concordance with reference assay. Finally, the Amplix platform appeared robust without any intersamples contamination.

Since misestimating of the plasma viral load could lead to inappropriate therapeutic management, the comparative performances of the assays, especially their ability to span the genetic diversity of HIV-1, need to be evaluated. This point is a major criterion to be considered for the development of assays able to amplify different HIV-1 subtypes. Indeed, recent data suggested viral load discrepancies between commercial quantitative assays, especially for the quantification of non-B subtype strains [16–18]. Indeed, several available techniques for HIV-1 RNA load were developed only based on subtype B [26, 27], which represent approximatively 10% of HIV strains worldwide. Subtype B is mainly found in Europe and northern countries while non-B subtypes are predominant in the world. In our hands, the Amplix kit targeting conserved* gag* and* LTR* regions of the HIV-1 genome was fully sensitive to detect and quantify the most prevalent strains of HIV-1 circulating in Central Africa, including CRF11_cpx, CRF01-AE, and subtype A, as well as the majority of non-B subtypes and CRFs of group M and also groups N and O.

The assay showed excellent correlations and concordance with reference to CAP/CTM TaqMan HIV-1 test v2.0. However, the mean absolute bias was +0.097 log copies/mL with limits of agreement from −0.657 to +0.851 log copies/mL by Bland-Altman analysis. This is in line with previous comparative studies testing multiple subtypes, discrepant samples identified by Bland-Altman analysis included those with lower viral loads, and non-B subtypes [14, 16, 28, 29]. There may be issues relating to detection of low levels of viremia with specific assays [28]. Furthermore, similar to the reference CAP/CTM TaqMan HIV-1 test v2.0, the Amplix HIV-1 quantitative real-time PCR kit is a highly sensitive dual-target assay for which the likelihood of proviral DNA amplification in addition to viral RNA may contribute to additional low-level quantitative signals. Finally, technological factors may furthermore account for some of the variation observed between assays and contribute to the significant absolute positive bias at nearly 0.1 log copies/mL with the Amplix HIV-1 quantitative real-time PCR assay.

Recently, intermediate high-throughput laboratory platforms for HIV RNA viral load have emerged [6]. Intermediate viral load platforms are expected to overcome some of the technological limitations to improve access to viral load as well as reducing the costs and may be particularly adapted in African settings for categories III and IV laboratories, according to the 2008 Maputo classification of laboratory facilities [9]. The main advantages of nucleic acid-based approaches include the following: many of the assays using these approaches have been evaluated and are well validated, the assays are available in quality-assured kits, and clinicians are comfortable interpreting the results. However, factors restricting access include the need for sophisticated laboratory capacity and instrumentation, along with training for laboratory technicians and well-functioning sample transport networks.

The HIV-1 RNA-based Amplix real-time PCR platform offered various advantages that make it equivalent to conventional systems in analytical terms and likely more adapted to common laboratory facilities currently existing in sub-Saharan Africa. First, the assay demonstrated its clinical usefulness for HIV-1 RNA viral load monitoring in HIV-1-infected patients. It required only 190 *μ*L of plasma, making it suitable for use on both children and adults. The assay has given excellent results for the monitoring of ART efficacy, as reported previously [30]. Second, the test has good feasibility and practicality. The entire Amplix platform, including extraction and amplification steps, did not need dedicated laboratory space, with physical separation between extraction and amplification rooms, which is necessary for standard classical PCR techniques. The Amplix distributor based in France is the direct supplier in the Central African Republic, and delivery time did not exceed more than 3 weeks. Importantly, the total duration of the analysis of one run is 3 hours (1 h 30 for extraction and 1 h 30 for amplification) on Amplix* versus* 6 hours (2 h 30 for extraction and 3 h 30 for amplification) for CAP/CTM TaqMan (Roche Molecular Diagnostics). The thermoresistant lyophilized format of the Amplix HIV-1 RNA quantitative reagents (Biosynex) may be particularly useful in tropical or subtropical areas. The capacity of the Amplix extraction system is flexible from 1 to 16 samples per run, a workflow fully adapted to intermediate laboratory facilities, whereas the CAP/CTM TaqMan platform realizes series of 4 to 72 samples per run, with significant consumption of controls and consumables such as S-tube used for extraction. Moreover, with one trained technician, 48 HIV-1 RNA clinical results could be obtained by Amplix platform within 4 h, including 1 h 30 for ARN extraction and 2 h 30 for amplification and lecture. Thus, this high-throughput platform required significantly less hands-on time than the conventional commercial assays and is expected to carry out around 10,000 HIV-1 RNA load per year.  Table 6 depicts the workflow, cost test, and equipment cost for the Amplix and CAP/CTM TaqMan platforms (Table 6). The quantitative Amplix HIV-1 RNA assay was as expensive as other commercial HIV-1 RNA kits from the laboratories participating to the Global Access Program for HIV viral load testing, created in partnership with UNAIDS, the Clinton Health Access Initiative (CHAI), the President's Emergency Plan For AIDS Relief (PEPFAR), and the Global Fund to fight AIDS, TB & Malaria (i.e., cost per test around 10 US $). Furthermore, the Amplix real-time PCR platform is multiparametric and offers numerous markers for HIV-related comorbidities, including hepatitis B and hepatitis C,* Mycobacterium* complex, and rifampicin resistance (detection of mutations of the* rpoB* gene of* Mycobacterium tuberculosis*). Finally, the automatic nucleic acids extraction system and the thermocycler of the Amplix real-time PCR platform are CE-IVD marked and available for sale in the European Union. The Amplix NG thermocycler is an open system and is not limited for use with specific reagents. Interestingly, its compact body design (21 × 48 × 31 cm) allows several thermocyclers controlled simultaneously by one computer to be placed closer together to increase productivity at minimal space requirements.

The disadvantages of the Amplix PCR platform are those common to all systems for HIV-1 RNA measurement such as seemingly expensive equipment cost (around $38,000 for the entire platform) and equipment maintenance.

In conclusions, our study shows that the HIV-1 RNA-based Amplix real-time PCR platform is sensitive and reliable for clinical monitoring of HIV-1 RNA viral load in HIV-1-infected children and adults, particularly adapted to intermediate laboratory facilities in sub-Saharan Africa.

## Figures and Tables

**Table 1:** Lower limit of detection of the HIV-1 RNA-based Amplix real-time PCR assay using the 3rd WHO HIV-1 subtype B international standard (National Institute for Biological Standards and Control) at 185,000 IU/mL (e.g., 5.04 log copies/mL), estimated at 28.1 HIV-1 RNA copies/mL (95% CI, 20.1–82.8 copies/mL)^*µ*^.

Level	HIV-1 RNA (copies/mL)	Number tested	Number positivity	Rate (%)
1	100	41	41	100
2	75	37	37	100
3	50	45	45	100
4	40	50	48	96
5	30	65	63	97
6	20	58	56	96
7	10	49	27	55

^*µ*^The lower limit of detection was calculated by PROBIT regression analysis using SPSS software.

**Table 2:** Repeatability and reproducibility of the HIV-1 RNA-based Amplix real-time PCR assay.

Panel	Target HIV-1 RNA (log copies/mL)	Number of determinations	Mean (SD) (log copies/mL) of HIV-1 RNA measured	Coefficient of variation (%)
Repeatability				
Third WHO standard	5.04	3	5.2 (0.3)	**5.7**
Low-positive pool	3.17	30	3.2 (0.2)	**7.7**
High-positive pool	6.33	30	6.5 (0.3)	**4.6**

Reproducibility				
Third WHO standard	5.04	3	5.3 (0.4)	**7.5**
Low-positive pool	3.17	30	3.3 (0.3)	**9.1**
High-positive pool	6.33	30	6.6 (0.4)	**6.0**

SD: standard deviation.

**Table 3:** Estimation of the accuracy of the HIV-1 RNA-based Amplix real-time PCR assay at nominal standards (20, 100, 1,000, 5,000, and 50,000 copies/mL), prepared from the AcroMetrix HIV-1 panel (applied Biosystems).

	Copies/mL	Log copies/mL (*v* ^*∗*^)	Mean of 5 replicates (*x* ^*∗∗*^)	Bias (%)^*∗∗∗*^	Mean bias (%)
Standard # 1	20	1.3	1.4	4.6	**3.4**
Standard # 2	100	2.0	2.0	4.0	
Standard # 3	1,000	3.0	3.1	2.6	
Standard # 4	5,000	3.7	3.8	2.7	
Standard # 5	50,000	4.7	4.8	2.9	

^*∗*^ *v*: expected target value from the AcroMetrix HIV-1 panel.

^*∗∗*^ *x*: mean of 5 replicates obtained by the HIV-1 RNA-based Amplix real-time PCR assay.

^*∗∗∗*^Bias (%) = Mean([*x* − *v*]/*v*) × 100.

**Table 4:** Ability of the HIV-1 RNA-based Amplix real-time PCR assay to quantify B/non-B subtypes HIV-1 group M, HIV-1 group N, and HIV-1 O using the 2nd WHO international reference panel preparation of various HIV-1 subtypes (National Institute for Biological Standards and Control).

HIV-1 group	Subtype HIV-1 group M	Lowest detectable concentration (copies/mL) (success rate > 95%)
M	A	23
M	B	19
M	C	26
M	D	25
M	A/E	21
M	F	29
M	G	24
M	AA-GH	26
N	—	27
O	—	24

**Table 5:** Detection of virological failure, that is, HIV-1 RNA load above 1,000 copies/mL according to the WHO [13], by HIV-1 RNA-based Amplix real-time PCR assay and the reference COBAS AmpliPrep/COBAS TaqMan HIV-1 test v2.0 in 215 HIV-seropositive clinical plasma samples from antiretroviral drug-experienced patients^£^.

		Reference HIV-1 RNA load (Roche Molecular Diagnostics)	
		<1,000 copies/mL	>1,000 copies/mL
Amplix HIV-1 RNA load (Biosynex)	<1,000 copies/mL	45	0
	>1,000 copies/mL	2	168

^*£*^The proportions of HIV-infected, antiretroviral drug-experienced patients diagnosed in virological failure were similar by the reference COBAS AmpliPrep/COBAS TaqMan HIV-1 test v2.0 (168/215 = 78.1%) and the HIV-1 RNA-based Amplix real-time PCR assay (170/215 = 79.1%) (NS by Fisher's exact test).

**Table 6:** Run duration (extraction plus amplification and lecture), number of samples per run, test cost and equipment cost for HIV-1 RNA-based Amplix real-time PCR assay, and the reference COBAS AmpliPrep/COBAS TaqMan HIV-1 test v2.0.

Platform	Run duration (*hour*)	Number of samples per run	Cost/test (*US $*)	Equipment cost (*US $*)^*∗*^
COBAS AmpliPrep/COBAS TaqMan (Roche Molecular Diagnostics)	6	176 per 8 hour-day continuous loading	16	COBAS TaqMan 48: 45,000–100,000 COBAS AmpliPrep: 80,000–150,000

HIV-1 RNA-based Amplix real-time PCR (Biosynex)	3	48 per 6 hour-day continuous loading	15	ExiPrep™ 16 Dx: #18,000–25,000 Thermocycler NG 48: #12,000–20,000

^*∗*^According to UNITAID 2015 [6], the cost per test in the Global Access Program for HIV viral load testing is around 10 US $.
